# Draft genome sequence of *Parabacteroides distasonis* SZCHProba001, isolated from feces of a 6-year-old boy

**DOI:** 10.1128/mra.00422-25

**Published:** 2025-08-25

**Authors:** Zhongsheng Zhu, Chuqing Zhang, Zili Liao, Jiaojie Guan, Xiuxiu Xie, Ziyuan Li, Boxian Lin, Hu Chen, Zeling Zhuang, Shiyun Meng, Yigui Zou, Wenwen Li, Mingjing Luo, Dongling Dai

**Affiliations:** 1 International Medical Center, Endoscopy Center and Gastroenterology Department Shenzhen Children’s Hospital, Shenzhen, China; 2 State Key Laboratory of Quantitative Synthetic Biology, Shenzhen Institute of Synthetic Biology, Shenzhen Institute of Advanced Technology, Chinese Academy of Sciences 85411 , Shenzhen, China; 3 Shenzhen Children’s Hospital, Southern University of Science and Technology 255310, Shenzhen, China; 4 The Chinese University of Hong Kong 407605, Shenzhen, China; 5 Shenzhen University Health Science Center 477382, Shenzhen, China; DOE Joint Genome Institute, Berkeley, California, USA

**Keywords:** *Parabacteroides distasonis*, genome, gut microbiome

## Abstract

*Parabacteroides distasonis* is a prevalent human gut bacterium. Here, we report the draft genome of *P. distasonis* SZCHProba001 isolated from a 6-year-old boy’s feces. The 5.03 Mb assembly (102 contigs) contains 4,099 predicted proteins and 76 RNAs. This resource aids research into *P. distasonis*' role in pediatric gut health.

## ANNOUNCEMENT

*Parabacteroides distasonis* is an anaerobic, gram-negative gut commensal with complex host interactions (1). In murine models, it demonstrated protective roles, including anti-inflammatory, anti-cancer, and metabolic benefits (2–6). Meanwhile, it has been implicated in human autoimmunity via molecular mimicry and shows reduced abundance in children with autism spectrum disorder (7, 8). This study presents the draft genome of *P. distasonis* strain SZCHProba001, isolated from a child’s fecal sample, to expand resources for comparative genomics and mechanistic studies of this species.

A fecal specimen from a 6-year-old male was self-collected into anaerobic preservation medium (0.05% L-cysteine hydrochloride and 20% glycerol in PBS) at home on 15 May 2024. It was transported to the lab within 2 h and maintained at −80°C until downstream procedures. Microbial cultivation was executed within a controlled anaerobic environment (gas mixture: 10% H₂, 5% CO₂, 85% N₂). For bacterial isolation, the specimen was aseptically plated onto Columbia blood agar (Detgerm, China) and subjected to 48 h incubation at 37°C. Homogeneous microbial colonies were isolated through three sequential subculturing steps, then identified by MALDI-TOF mass spectrometry (autoflex maX, Bruker, USA). A *P. distasonis* colony was picked and streaked onto a fresh agar plate for another 48 h culture. The purified clones were scraped off for DNA extraction using the Wizard Genomic DNA Purification Kit (Promega, USA). Library preparation for sequencing was performed with the TruePrep Flexible DNA Library Prep Kit for Illumina (Vazyme, China). Final libraries were sequenced on the Illumina NovaSeq 6000 system, generating 150 bp paired-end reads.

Quality control and filtering of raw reads were performed with Fastp (v0.23.4) (9). Genome assembly was then carried out with Unicycler (v0.5.0) (10), and the assembly quality was subsequently evaluated using QUAST (v5.2.0) (11). The genome completeness, determined with CheckM (12), reached 99.42%. The *P. distasonis* SZCHProba001 genome spans 5,025,565 bp with a 45% G + C content, distributed over 102 contigs, and exhibits an N_50_ of 150,747 bp. Sequencing produced 3.12 million paired-end reads of 150 bp each, resulting in a 93× coverage depth. Annotation via the NCBI Prokaryotic Genome Annotation Pipeline (v6.10) (13) identified 4,099 protein-coding sequences, 67 tRNAs, 7 rRNAs, and 2 ncRNAs. Average nucleotide identity (ANI) analysis by Genome Taxonomy Database Toolkit (GTDB-Tk, v2.4.0, database version r220) (14) with the --classify_wf workflow and FastANI (v1.33) (15) revealed a 97.69% ANI with the type strain *P. distasonis* ATCC 8503 (accession GCF_000012845.1), 94.2% with *P. distasonis_*A (accession GCF_004793765.1), and 90.43% with *Parabacteroides sp*. (accession GCA_028689545.1). No plasmids were detected using PlasmidFinder (v2.1.6) (16), and PHASTEST (17) did not identify any prophage regions. Additionally, whole-genome taxonomic classification performed with GTDB-Tk confirmed the isolate SZCHProba001 as *P. distasonis*. Default parameters were used, except where otherwise noted. This *P. distasonis* SZCHProba001 genome contains putative genes involved in aromatic amino acid transport and metabolism (*trpA*, *trpB*, *trpS, pheT,* and *pheS*) and vitamin biosynthesis and transport (*bioB, btuBCD,* and *cobDT*) (Table 1). It also contains the colistin resistance gene *emrA* and the tetracycline resistance gene *tetQ* (Table 1).

**TABLE 1:** Key *P. distasonis* SZCHProba001 genomic elements^*a*^

Gene	Type	Product
*arnA*	AMR	Bifunctional polymyxin resistance protein ArnA
*emrA*	AMR	Colistin resistance protein EmrA
*fsr*	AMR	Fosmidomycin resistance protein
*tetQ*	AMR	Tetracycline resistance ribosomal protection protein TetQ
*tyrS*	AATM	Tyrosine--tRNA ligase
*ywqE*	AATM	Tyrosine-protein phosphatase YwqE
*ptk*	AATM	Tyrosine-protein kinase ptk
*trpA*	AATM	Tryptophan synthase alpha chain
*trpB*	AATM	Tryptophan synthase beta chain
*trpS*	AATM	Tryptophan--tRNA ligase
*pheT*	AATM	Phenylalanine--tRNA ligase beta subunit
*pheS*	AATM	Phenylalanine--tRNA ligase alpha subunit
*bioB*	VBAT	Biotin synthase
*btuB*	VBAT	Vitamin B12 transporter BtuB
*btuC*	VBAT	Vitamin B12 import system permease protein BtuC
*btuD*	VBAT	Vitamin B12 import ATP-binding protein BtuD
*cobD*	VBAT	Threonine-phosphate decarboxylase (Vitamin B12 biosynthesis)
*cobT*	VBAT	Nicotinate-nucleotide--dimethylbenzimidazole phosphoribosyltransferase (Vitamin B12 biosynthesis)
*nadC*	VBAT	Nicotinate-nucleotide pyrophosphorylase [carboxylating] (Vitamin B3 biosynthesis)
*nadD*	VBAT	Nicotinate-nucleotide adenylyltransferase (Vitamin B3 biosynthesis)
*pnuC*	VBAT	Nicotinamide riboside transporter PnuC (Vitamin B3 biosynthesis)

^*a*^ AMR, antimicrobial resistance; AATM, Aromatic amino acids transport and metabolism; VBAT, Vitamin biosynthesis and transport.

## Data Availability

The genomes have been deposited in the NCBI under BioProject PRJNA1250271 and BioSample SAMN47936688. Raw sequence reads are available at the NCBI Sequence Read Archive (SRA) under accession number SRR33208112. The assembled Genomegenome has been deposited at GenBank under the accession JBNCMM000000000.
